# Building consensus on key priorities for rural health care in South Africa using the Delphi technique

**DOI:** 10.3402/gha.v6i0.19522

**Published:** 2013-01-24

**Authors:** Marije Versteeg, Lilo du Toit, Ian Couper

**Affiliations:** 1 Rural Health Advocacy Project, Centre for Rural Health, Faculty of Health Sciences, University of the Witwatersrand, Johannesburg, South Africa; 2 School of Public Health, Faculty of Health Sciences, University of the Witwatersrand, Johannesburg

**Keywords:** rural health, priorities, challenges, Delphi technique, health systems, leadership, management

## Abstract

**Background:**

South Africa is currently undergoing major health system restructuring in an attempt to improve health outcomes and reduce inequities in access. Such inequities exist between private and public health care and within the public health system itself. Experience shows that rural health care can be disadvantaged in policy formulation despite good intentions. The objective of this study was to identify the major challenges and priority interventions for rural health care provision in South Africa thereby contributing to pro-rural health policy dialogue.

**Methods:**

The Delphi technique was used to develop consensus on a list of statements that was generated through interviews and literature review. A panel of rural health practitioners and other stakeholders was asked to indicate their level of agreement with these statements and to rank the top challenges in and interventions required for rural health care.

**Results:**

Response rates ranged from 83% in the first round (*n*=44) to 64% in the final round (*n*=34). The top five priorities were aligned to three of the WHO health system building blocks: human resources for health (HRH), governance, and finance. Specifically, the panel identified a need to focus on recruitment and support of rural health professionals, the employment of managers with sufficient and appropriate skills, a rural-friendly national HRH plan, and equitable funding formulae.

**Conclusion:**

Specific policies and strategies are required to address the greatest rural health care challenges and to ensure improved access to quality health care in rural South Africa. In addition, a change in organisational climate and a concerted effort to make a career in rural health appealing to health care workers and adequate funding for rural health care provision are essential.

South Africa has a sizeable rural population, comprising 43.6% of its 49 million inhabitants (1). The majority of rural people are poor and rely almost entirely on the public health system (2). The current health system is curative in focus, high but inequitable spending on health, the country is facing a quadruple disease burden (consisting of HIV & AIDS and tuberculosis (TB), chronic diseases, injuries, and maternal and child mortality), and health outcomes are poor. These are the main factors that have led the South African Government to embark on a ‘total overhaul’ of the system (3) to meet its constitutional mandate to provide access to quality health care for all South Africans (4).

Rural communities are amongst the most disadvantaged in terms of accessing quality health care. A child living in the Eastern Cape province (predominantly rural) is more than twice as likely to die in its first year of life than a child from the Western Cape (containing large urban areas), while a person with TB in the Gauteng province (predominantly urban) has a 19.9% higher chance of being cured than a person with TB in the North West province (predominantly rural) (5). In 2007, South African infant mortality rates were found to be 71.2 per 1,000 live births in rural areas compared to 43.2 in urban areas (6). Other developing countries show similar inequities in urban–rural health outcomes (7). In the same year (2007), India reported 62 deaths per 1,000 live births in rural areas compared to 39 in urban areas (8). The United Nations notes that, globally, children in rural areas are at greater risk of dying, even where overall child mortality is low (9).

Recent policy developments in South Africa include the primary health care (PHC) re-engineering strategy (10), a new national human resources for health plan (11), and the proposed introduction of a national health insurance (NHI) (12), all of which aim to achieve quality health care access for all. They have been received with much positive anticipation by rural health practitioners. However, experience has made these practitioners cautious in their optimism. This stems from past policy decisions which, unintentionally, impacted negatively on or were difficult to implement in rural settings. Specific examples in the South African context include the closing of rural hospital-based nursing colleges and the introduction of a salary structure for public sector health professionals that rewards specialisation in urban centres (13).

A special focus on rural health has not been the norm in the South African health policy landscape. Yet, globally, there is a trend among countries with large rural populations to develop targeted interventions for rural health care. For instance, the Indian government established a rural health mission which aims to improve access to quality health care for people residing in rural areas (8), while Australia adopted a rural health strategy in 1994 (14). The UK government introduced the concept of ‘rural-proofing’ of all domestic policies, including health policies, and made it a mandatory part of the policy-making process (15). This concept of ‘rural-proofing’ involves ensuring that all relevant policies are examined to determine whether they would or could have a different impact in rural areas from elsewhere, because of the unique characteristics of rural areas, and are adjusted, where necessary, to reflect rural needs and to ensure that public services are equally accessible to a rural community (16).

The rationale for arguing that special policy attention be given to rural health care is based on the disparities in access and health outcomes between rural and urban settings (2). A World Bank report on geographical imbalances in the distribution of health workers indicated that rural health facilities lack the required numbers and skills mix needed in most sub-Saharan countries (17). Although the situation is worse in other African countries, it is nevertheless true for South Africa. The WHO recommends a health worker density of 2.28 health workers per 1,000 population as a minimum to achieve health-related Millennium Development Goals (9). South Africa is below this minimum nationally and even more so in rural areas; in 2010 in the public sector there were 0.29 doctors and 1.35 nurses per 1,000 population nationally, compared to 0.24 doctors and 0.81 nurses per 1,000 in the North West province (predominantly rural) (18).

The design of health policies can (intentionally or unintentionally) leave out the interests of certain groups. The formulation of public health policy is often a complex issue, and the groups influencing it in particular contexts may not necessarily represent the interests of the marginalised (19). In South Africa, there has been insufficient lobbying for the needs and interests of rural populations. This is reflected not only in past policy decisions, such as those referred to earlier, but also in the critical issue of budget allocations. Although South Africa spends 8.6% of its gross domestic product (GDP) on health, more than any other African country, the majority of this is spent in the private health care sector, which is accessible only to 14% of citizens. Only 3.5% of GDP is spent in the public health sector, of which rural areas remain the most under-resourced. Health expenditure per capita is highest in the two most urban provinces Gauteng and Western Cape (108.9% and 106.0% of the average), and as low as 81.6% in the rural North West province (20). Health expenditure on PHC in 2010/2011 was R404 per person (about US$45) in the most deprived districts, which are all rural, versus R584 (about US$66) in the least deprived districts, which are mostly urban (21). Thus, provinces with the greatest burden of disease and the least economic resources, yet still with large populations, receive the smallest share of funds for public health care (22). Health care workers in rural areas have also commented that their interests, and those of the communities they serve, are not sufficiently heard (23).

Given this background of inequity, a health system in transition and past experiences of policy-making that was not rural-proofed, we sought to understand what rural health workers believe to be the key challenges and priority interventions required for rural health care. The intention was thus to give rural health care workers a voice to influence policy discussions.

For the purpose of this research, we defined rural health care as provision of health services to areas outside of metropolitan centres where there is not ready access to specialists, intensive and/or high technology care, and where resources, both human and material, are lacking. This service may be within hospitals, health centres, clinics, within homes in communities, or independent practices. It is best provided by a team of health care workers and is based on the principles of PHC (24).

## Objectives

The aim of the research was to obtain consensus among a group of people involved in rural health care regarding the biggest challenges and most important priorities for rural health care delivery in South Africa. The purpose of this article is to make this information available to policy makers to advocate for equitable access to health care as well as improved health outcomes for rural citizens.

## Methods

The Delphi method was used to obtain consensus from a panel of experts with a wide variety of opinions and views on the challenges and priorities for rural health care.

### Rationale

Key characteristics of the Delphi process include (a) structuring information flow with the aim of focusing on relevant content; (b) facilitating regular feedback by allowing participants to revise their opinions at any given time; and (c) guaranteeing anonymity, thus allowing participants the privacy to express opinions that may be contrary to the group's or leader's view. Participants can also suggest new issues for consideration at any stage (25). Although it originated as a predictive methodology, it is often used in exploring issues in a more normative sense (how *should* it be, apart from how it *is*) (25). The method is particularly useful within the rural context. As time and distance were not limitations for participation, participants from a variety of groups and contexts took part (26), and it enabled the research team to obtain consensus from a range of stakeholders with differing backgrounds and perspectives, from all over South Africa.

### The Delphi panel

Fifty-three panellists across seven out of nine provinces in South Africa consented to participate in the Delphi process. Participants were selected purposively on the basis of either frontline or policy level expertise in relation to rural health care delivery and included senior representatives from provincial departments of health, hospital managers, clinical managers, family physicians, medical officers, nursing professionals, rehabilitation professionals, medical specialists, such as an obstetrician and paediatrician, policy experts, and activists in the field of human rights and health. Most respondents were identified directly by the researchers. In addition, suitable health care workers in remote areas were approached on the basis of recommendations from other rural health care workers.

### Data collection

Two steps were followed to generate a list of issues to be considered by the Delphi panel. We conducted in-depth interviews with three rural health experts, who were involved in rural practice in three different provinces, namely KwaZulu-Natal, Western Cape, and Mpumalanga. This information provided the content and scope for a literature review. Combining these, a list of statements was drawn up, organised into six themes aligned to the WHO health system building blocks: service delivery; health workforce/HRH; information; medical products, vaccines and technology; financing; and leadership and governance (27).

In a process comprising three questionnaire rounds, participants were asked to:

1. Indicate their level of agreement with 153 statements, representing challenges or priority interventions, on a Likert-scale from 1 to 5 (strongly disagree to strongly agree);
2. Introduce new challenges and priorities if applicable; and
3. Rank the top five challenges and priorities.

**Fig. 1:**
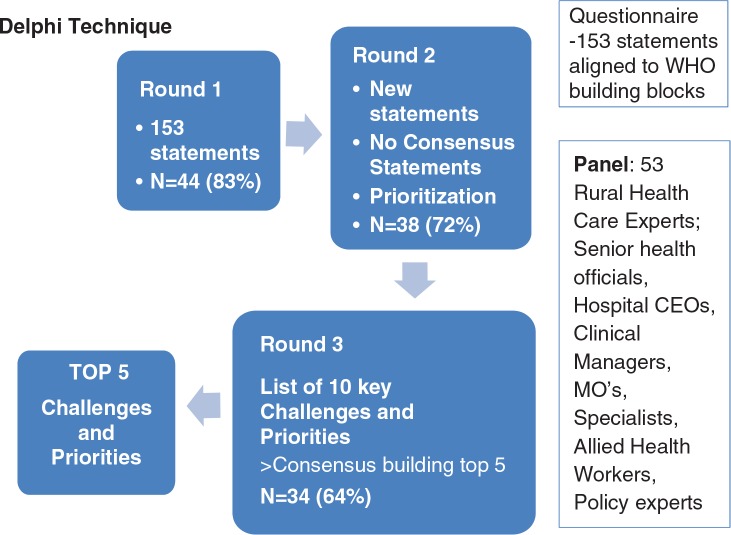
Delphi process to determine key challenges and priorities for rural health care.

In the second and third round, a weighted scoring system was applied (i.e. a statement that was ranked as the top priority received a score of 5 and the one ranked as the fifth priority was assigned a score of 1). In the final (third) round, the top 10 challenges and the top 10 priorities were listed, and participants were asked to arrange them in their preferred order of importance. Figure 1 illustrates the research process.

The protocol was approved by the Human Research Ethics Committee (Medical) of the University of the Witwatersrand. Participation was voluntary and consent forms were signed prior to participation. The confidentiality of participants was maintained.

## Results

Participation in this process was good, with response rates ranging from 83% in the first round (*n*=44) to 64% in the final round (*n*=34) (Table 1). Participants frequently provided lengthy motivations for their choices, indicating a high level of engagement.

**Table 1:** Participation of respondents by sector

	Round 1		Round 2		Round 3	
			
Position	Number	%	Number	%	Number	%
Academic	6	14	5	12	5	15
Allied health	6	14	6	14	6	18
Civil society	6	14	6	14	3	9
Manager	6	14	6	14	5	15
Medical officers	19	43	19	44	14	41
Nurse	1	2	1	2	1	3
Total	44	100	43	100	34	100

Table 2 presents the top five challenges that were identified through this process and puts them alongside the top five priorities.

**Table 2:** Top five challenges alongside top five priorities

	Statements and their rankings under ‘challenges’			Statements and their rankings under ‘priorities’		
		
Theme	Statement	Ranking round 2	Ranking round 3	Statement	Ranking round 2	Ranking round 3
Governance & leadership	People are appointed to senior posts in hospitals, district offices, and provincial departments of health without requisite knowledge, skills, and experience.	3rd	1st	Hospital/medical managers should be employed based on the appropriate skills and experience.	1st	2nd
HR for health	Comprehensive and equitable rural health care is hampered by the mal-distribution of health workers between urban and rural sectors, private and public sectors, and at different levels (tertiary hospital to PHC clinic).	1st	2nd	There is a need to focus on how to recruit, retain, and support senior health care professionals in rural hospitals for the long term.	2nd	1st
HR for health	Provincial HR departments are often weak, causing a variety of problems such as a lack of a sense of urgency in dealing with important matters, namely recruitment of professionals, including advertising of posts.	4th	3rd	There is a need for the development and implementation of a national HR plan that is relevant to the rural health care context.	3rd	3rd
Finance	Budget cuts and frozen medical positions take place across the board and do not take into account the often fragile positions of already under-resourced rural hospitals.	2nd	4th	Equitable funding formulae need to be designed for the financing of hospitals based on the local burden of disease, staffing needs, the costing of services, and equity principles.	7th/8th	4th
Governance & leadership	There is a lack of work ethic and value systems among health care workers and management that hamper a motivating climate in health facilities.	5th/6th	5th	District managers should be employed based on the appropriate skills and experience.	4th	5th

The appointment of people to senior positions without the requisite knowledge and skills was the highest ranked challenge identified by the panel, followed by the mal-distribution of health care workers. The remainder of the top challenges were the weak role of provincial health departments in dealing with urgent human resource (HR) matters such as recruitment of health professionals, budget cuts, and frozen medical posts that do not take into account the fragile positions of already under-resourced rural hospitals, and the lack of a work ethic and value systems among health care workers and management, hampering a motivating climate in health facilities.

The interrelatedness of some of the challenges and their impact on delivery of rural health care was reflected in the comment of one panellist:

Senior and managerial posts should be filled based on suitability of the candidate in terms of knowledge, experience and vision. Placing inappropriate people in senior posts just to fill a gap leads to demoralisation of people having to work with/under them, a breakdown of service standards, inefficiency and poor service delivery.

The intervention that was ranked as the highest priority was to focus on recruiting and retaining health care workers in rural hospitals. Appointing people to senior posts in hospitals, district offices, and provincial departments of health with the requisite knowledge, skills, and experience was the second highest priority, linking well with the challenges identified above. The remaining priority interventions ranked in the top five were the need to develop and implement a national HRH plan that is relevant to rural health, the need to design equitable funding formulae, and the need to employ district managers based on appropriate skills and experience.

In terms of the critical skills required by health managers (priorities three and five), many respondents argued that people skills are of equal importance to health management skills. A respondent stated this as follows:

There is a very impressive middle manager at a little hospital in my area. (…) He notices issues that are happening on the ground. And he listens to people. We had a crisis in finding a bed for a lady with MDRTB [Multiple Drug Resistant TB]. The nurses had tried to do their best. He called the whole team in, got everyone to discuss how best to solve this problem, then instructed the matron to turn an empty antenatal care ward into a temporary MDR ward. The whole team felt so supported. He thanked the nurses who had made some temporary plans (and didn't bomb on them!) and told them that they were showing true compassion for their patients. He is also prepared to move into the not-the nicest office, in order to make more clinical space!

As part of the solution to the HRH shortage and challenges at management level, a panellist made the following suggestion:

We are not using doctors enough in management to plan and oversee the health systems issues. … If nurses are well trained and well supported, they are able to take over many of the basic clinical roles of doctors. On the other hand, we need to rely more on doctors with their good overview and understanding of the whole health system in planning and prioritising care.

## Discussion

It is evident that the top five priority interventions for rural health care presented in Table 2 largely reflect the top five challenges: the opinions of the panel coalesce around governance, leadership, and HRH. Three of the priorities identified were introduced by participants in round 1, and their importance was confirmed in subsequent rounds. Although there were variations in the ranking of the top challenges and priorities, the themes were common. These are discussed below.

### Human resources for health

Human resource issues, in the opinion of the expert panel, emerged as the biggest challenge to improve comprehensive, quality health care for rural citizens. This is in line with the assertion by the WHO that the failure to mobilise an effective health workforce is the most important obstacle to improving the performance of health systems and, in turn, achieving key health objectives (27). Although three different WHO building blocks (health workforce, finance, leadership and governance) feature in the top five challenges, it is notable that they all have links to human resources for rural health.

Given the low doctor-to-population ratio in the rural provinces of South Africa, Limpopo being worst affected, followed by Mpumalanga, the Eastern Cape, and North West (28), it is not surprising that the Delphi panel identified the mal-distribution of health care workers as the second greatest challenge. While this problem is a global one, there are strategies that can increase access to health workers in rural and remote areas; as outlined in the 2010 WHO global policy recommendations. These include educational strategies (for which there is the most evidence), regulation, financial incentives, and personal and professional support (29). Appropriate selection of students and training of health professionals in rural areas have been shown to be interventions that can redress the inequitable distribution of health workers (30). In the Philippines, the Zamboanga School of Medicine in rural Southern Mindanao province provides a successful example; more than 90% of its graduates are continuing their training and clinical practice within the region, in a context where the majority of medical graduates leave the country (31).

This mal-distribution of the health workforce is also found *within* rural districts, which results from the lack of effective recruitment and retention strategies as well as the lack of staffing norms and needs-based HRH allocations (28). This is further compounded by weak provincial HR departments (third greatest challenge). Although it is difficult to fill posts in rural areas, this is aggravated by the long delays in filling posts even when interested and suitable candidates are available (28).

It is interesting that the financial compensation of health care workers did not arise as a key challenge, rather the underfunding and rationalisation of health spending without regard to the often fragile situation of rural health facilities (fourth greatest challenge) was considered a key challenge. This links to the fourth priority, which refers to the underfunding of rural health care and the need for equitable formulae. Here, it is important to note that rural hospitals are often more expensive to run than urban hospitals due to their lower economy of scales (less dense population being serviced), with the great distances between people and services raising both demand and supply costs. Indeed, many hospitals in remote areas exist to improve access to services and to redress inequity rather than the high population numbers in the area (which would bring down the average cost per population). The argument that rural health services need to be treated differently in relevant aspects to ensure equitable outcomes (32) forms the basis for policy making that include a specific rural health focus in a number of countries, such as the United States (33), Canada (34), and Australia (35).

The biggest losers as a result of staff shortages, low morale, frozen posts, and poor governance in rural areas are rural health care users. One testimony to this is the high number of avoidable and modifiable factors in maternal and child mortality ratios at health system level, with 22% of child deaths related to administrator action, such as lack of senior doctors and nurses, and 53% related to health care provider action, such as poor assessment and management in hospitals (36).

### Governance and leadership

The first and the fifth greatest challenges are inter-related and concern governance and leadership matters that have a direct bearing on the recruitment and retention of the rural health workforce, namely the quality of management and the organisational culture in health facilities. Although weak HR management affects the public health system across the board, the impact on rural facilities is most devastating due to their disproportionate state of fragility. According to various Delphi panellists, the appointment of senior managers that do not have the requisite training and skills within such systems can lead to uninformed decision-making, a lack of urgency in dealing with crisis situations, a poor work ethic, poor work relations, and low staff morale.

Globally, poor management and a lack of leadership skills are understood to be key drivers of HR problems in the broader health system, both in the developed and developing world (37–40). It is well-documented that good leadership inspires good performance by junior staff and vice versa. This is important because poor work ethic was identified by the panellists as a problem not only among management but also among health care workers. Good leadership includes the development of a joint vision; building adherence; strengthening accountability; planning, implementing, and monitoring HRH policies; and transparency in decision-making (41).

The South African government has recently announced plans for academic programmes to improve management and leadership skills. Although this is one important part of the solution, such programmes will have limited success when implemented within hierarchical organisational structures that discourage accountability (42). A negative organisational climate discourages skilled and committed people from staying in their positions over the longer term. Ultimately, the link between a system that fails to respond to contextual health care needs and an organisational culture that does not support visionary leadership and responsiveness needs to be made explicit (43). In various contexts, the argument is being made for decentralisation and deconcentration of health care services as a means of creating more flexible and contextually responsive health systems (44). This should be paired with local accountability. As a Delphi participant commented:

Hospital and medical managers should be able to spend their budgets without central approval at every step of the way, and then be held accountable for what they spend.

Rural health care needs vision-driven, capable managers who act with a sense of urgency, who are not only role models to their own staff but who also get the support from their superiors to effect changes on the ground that are specific to their districts, facilities, and communities (45). Such examples do exist in rural hospitals and these managers need to be nurtured and celebrated. This demands a change in organisational culture in many health settings, towards a climate where advocacy for patients’ rights is rewarded and acted on, and where the vision of the Department of Health of a caring and humane society in which all South Africans have access to affordable, good quality health care is the common goal of all working in the health system.

### Limitations

There were some limitations to this study. The panel was dominated by rural doctors, with fewer facility managers or nurses. This was both a result of the response rate and the sampling strategy and may have introduced some bias. Although the researchers sought to include health professionals of different backgrounds, our access to insightful, practising rural doctors resulted in this group being over-sampled. Furthermore, during the recruitment stage, many people were approached but only 53 respondents consented to take part. At the same time, analysis of the responses by doctors compared to the other categories revealed a large degree of consensus. For instance, 93% of respondents in round 1 felt that an HRH plan relevant to the rural context was either a ‘key priority’ or a ‘priority to a large extent’. Because of the small numbers of individuals in this study, a more detailed analysis by different professional groups could not be undertaken.

The Delphi technique is often criticised as the results may be biased towards the views of the facilitator (who ultimately collates feedback from participants) and that opinions, even if shared by many, may not be based in fact (26). In this particular study, it is possible that people with similar problems gave their opinions, though we sought to make the panel as diverse as possible. Such a study could be more useful if all provinces and all levels of health workers had an equal chance of being sampled. However, the findings were also consistent with issues that have been well described in the current policy dialogue in South Africa and give voice to front-line rural practitioners who are not always consulted on policy-making.

## Conclusion

Many rural health workers fear that there is little scope for positive change in rural facilities, which are at risk of remaining understaffed and poorly managed, thus entrenching existing inequities. This highlights the need, globally, for public health policy developments to be reviewed in terms of their possible impact on rural areas. The goal of this article was to present the consensus views of key informants and health experts on priority issues in rural health care. It is hoped that it can be used as an advocacy and lobbying tool to promote focussed policy development for rural health care.

To respond to the greatest rural health challenges, governments need to develop well-targeted rural health strategies that address HR distribution, financing, and governance issues and also ensure rural-proofing of new policy initiatives to prevent any negative impact on rural health care. In the 2011 HRH plan for South Africa (11), the National Department of Health identified the issue of management and leadership in the health sector as the first priority and also, for the first time, included a detailed chapter on HRH for rural health. Promising as these developments are, the underlying conditions for change must include equitable financing for rural health care provision, management appointments based on appropriate skills and experience, and transforming the organisational culture.
